# Sociocultural and Socioeconomic Influences on Type 2 Diabetes Risk in Overweight/Obese African-American and Latino-American Children and Adolescents

**DOI:** 10.1155/2013/512914

**Published:** 2013-05-13

**Authors:** Rebecca E. Hasson, Tanja C. Adam, Jay Pearson, Jaimie N. Davis, Donna Spruijt-Metz, Michael I. Goran

**Affiliations:** ^1^Schools of Kinesiology and Public Health, University of Michigan, 1402 Washington Heights, 2110 Observatory Lodge, Ann Arbor, MI 48109, USA; ^2^Department of Human Biology, Maastricht University, Maastricht, The Netherlands; ^3^Sanford School of Public Policy, Duke University, Durham, NC 27708, USA; ^4^Department of Nutritional Sciences, University of Texas Austin, Austin, TX 78712, USA; ^5^Department of Preventive Medicine, Keck School of Medicine, University of Southern California, Los Angeles, CA 90033, USA

## Abstract

*Purpose*. It is unclear whether sociocultural and socioeconomic factors are directly linked to type 2 diabetes risk in overweight/obese ethnic minority children and adolescents. This study examines the relationships between sociocultural orientation, household social position, and type 2 diabetes risk in overweight/obese African-American (*n* = 43) and Latino-American (*n* = 113) children and adolescents. *Methods*. Sociocultural orientation was assessed using the Acculturation, Habits, and Interests Multicultural Scale for Adolescents (AHIMSA) questionnaire. Household social position was calculated using the Hollingshead Two-Factor Index of Social Position. Insulin sensitivity (S_I_), acute insulin response (AIR_G_) and disposition index (DI) were derived from a frequently sampled intravenous glucose tolerance test (FSIGT). The relationships between AHIMSA subscales (i.e., integration, assimilation, separation, and marginalization), household social position and FSIGT parameters were assessed using multiple linear regression. *Results.* For African-Americans, integration (integrating their family's culture with those of mainstream white-American culture) was positively associated with AIR_G_ (*β* = 0.27 ± 0.09, *r* = 0.48, *P* < 0.01) and DI (*β* = 0.28 ± 0.09, *r* = 0.55, *P* < 0.01). For Latino-Americans, household social position was inversely associated with AIR_G_ (*β* = −0.010 ± 0.004, *r* = −0.19, *P* = 0.02) and DI (*β* = −20.44 ± 7.50, *r* = −0.27, *P* < 0.01). *Conclusions.* Sociocultural orientation and household social position play distinct and opposing roles in shaping type 2 diabetes risk in African-American and Latino-American children and adolescents.

## 1. Introduction

Type 2 diabetes and prediabetes have emerged as significant health issues in overweight/obese African-American and Latino-American pediatric populations in the United States (US). Data from the SEARCH for Diabetes in Youth Study indicate that incidence rates for type 2 diabetes were three times higher in African-Americans and Latino-Americans aged 15–19 years compared to non-Latino whites [1]. The Studies to Treat or Prevent Pediatric Type 2 Diabetes also reported a larger proportion of African-American and Latino-American children and adolescents with high fasting insulin levels compared to their non-Latino white counterparts (29.3%, 44.3%, and 20.5%, resp.) [2]. This ethnic disparity in diabetes and prediabetes has been linked to more severe insulin resistance and pancreatic beta-cell dysfunction in these ethnic minority children and adolescents [3].

While several behavioral mechanisms have been proposed to explain the increased diabetes risk in African-American and Latino-American children and adolescents [4, 5], research investigating the role of sociocultural factors (i.e., cultural attitudes, beliefs, and behaviors) is limited. With the rapid increase in cultural diversity of the US, African-American and Latino-American cultures are quickly becoming a part of mainstream American culture, evolving within the US, while simultaneously integrating aspects of different African and Latin American cultures [6]. These alternative cultures have aspects that are uniquely shaped by historical, social, political, and economic forces present in the US [7]. Consequently, African-American and Latino-American children and adolescents who come of age in the US, a multicultural society, interact with people from different cultural backgrounds which can lead to an interchange of cultural attitudes, beliefs, and behaviors [6].

Unger et al. [8] have argued that ethnic minority children and adolescents who interact with non-Latino whites may chose to adopt one of four sociocultural orientation patterns: (a) integration—combining aspects of their family's culture with aspects of mainstream American culture; (b) assimilation—replacing their family's culture with mainstream American culture; (c) separation—retaining their family's culture while rejecting mainstream American culture; or (d) marginalization—becoming alienated from both cultures [8]. This approach to conceptualizing sociocultural orientation is unique in that it emphasizes the psychological aspects of culture rather than assessing proxy indicators such as language use, nativity, and time in the US [9]. 

The limited number of studies assessing the influence of sociocultural orientation on type 2 diabetes risk suggests that, for African-Americans, integrating into mainstream American culture while retaining aspects of their own family's culture is inversely associated with diabetes risk through health-related behaviors [10]. The literature for Latino-Americans however is conflicting. With one notable exception [11], much research suggests integrating and/or assimilating into the mainstream American culture is positively associated with obesity [12–14] and suboptimal dietary choices [5, 15–17], whereas separation from mainstream American culture is positively associated with increased insulin resistance [11]. Although the influence of sociocultural factors on subsequent diabetes risk in African-Americans and Latino-Americans is striking [18], these findings may be confounded by socioeconomic position [19].

Sociocultural attitudes, beliefs, and behaviors are heavily influenced by an individual's socioeconomic environment [19]. Ethnic minority children and adolescents who reside in low socioeconomic households may be more likely to be segregated from non-Latino whites both at school and in their neighborhood [20] providing less exposure to a multicultural environment and limited sociocultural options. In contrast, ethnic minority children and adolescents living in middle-to-high socioeconomic households are more likely to live in racially mixed neighborhoods and/or attend predominantly white schools [20] thereby increasing interaction with individuals from different cultural backgrounds and expanding sociocultural orientation options. Despite the well-characterized relationships between sociocultural orientation, socioeconomic position, and type 2 diabetes risk [21], few researchers have attempted to disentangle these associations to better understand the increased diabetes risk reported in minority children and adolescents. Therefore, the primary objective of this study was to examine the independent relationships between sociocultural orientation, household social position, and type 2 diabetes risk in overweight/obese African-American and Latino-American children and adolescents. It was hypothesized that, for both minority groups, low household social position would be associated with increased diabetes risk defined as decreased insulin sensitivity (S_I_), decreased acute insulin response (AIR_G_), and decreased disposition index (DI) derived from a frequently sampled intravenous glucose tolerance test. Independent of household social position, integration (combining aspects of one's family's culture with aspects of mainstream American culture) would be associated with lower diabetes risk in overweight/obese African-American children and adolescents, whereas this same sociocultural adaptive style would be associated with increased diabetes risk in Latino-Americans.

## 2. Methods

All participants met the following inclusion criteria: age- and gender-specific BMI ≥ 85th percentile, African-American or Latino-American ethnicity (self-report and based on all four grandparents being of the same ethnic group as the child in the study), and between the ages of 8–18 years. Prior to any testing, informed written consent and assent were obtained from the participants and parents. All studies were approved by the University of Southern California Institutional Review Board. 

### 2.1. Procedures

Participants arrived at the General Clinical Research Center (GCRC) where a licensed pediatric health care provider conducted a medical/family history and physical examination which included an assessment of Tanner stage [22, 23]. Body composition was measured by air displacement plethysmography (BodPod; Life Measurement Instruments, Concord, CA). Total dietary intake was assessed with three-day dietary records given to participants to complete at home, which were later returned to research staff for nutritional analysis. Three-Day Physical Activity Recall was used to assess self-reported physical activity [24]. 

A frequently sampled insulin-modified intravenous glucose tolerance test (FSIGT) was used to assess type 2 diabetes risk [25]. Fasting plasma insulin and glucose concentrations were used to estimate insulin resistance using homeostasis model assessment (HOMA-IR), which was calculated as HOMA-IR = [(FPI × FPG)/22.5] [11]. Plasma collected during the FSIGT was analyzed for glucose and insulin, and values were entered into the MINMOD Millennium 2003 computer program (version 5.16, Bergman, USC) to calculate S_I_, AIR_G_, and DI. S_I_ was defined as the net capacity for insulin to promote the disposal of glucose and to inhibit the endogenous production of glucose. AIR_G_ was defined as the area under the plasma insulin curve between 0 and 10 minutes. DI, an index of beta-cell function, was calculated as the product of AIR_G_ and S_I_. Detailed descriptions of the FSIGT methods and protocols used in this study have been previously published [26–28].

During the GCRC visit, participants also completed a questionnaire regarding sociocultural orientation and parents of participants answered questions regarding parental educational attainment and occupational rank. Sociocultural orientation was assessed using the Acculturation, Habits, and Interests Multicultural Scale for Adolescents (AHIMSA) questionnaire [8]. The eight items on the AHIMSA as well as the four response items are listed in Table 1. The AHIMSA responses were divided into four sociocultural orientation subscales: “the US” was categorized as assimilation (Cronbach alpha: 0.79); “the country my family is from” was categorized as separation (Cronbach alpha: 0.68); “both” was categorized as integration (Cronbach alpha: 0.79); and “neither” was categorized as marginalization (Cronbach alpha: 0.50). For the African-American participants, the AHIMSA responses for assimilation and separation were modified to read “white-American culture” and “my family's culture,” respectively (Cronbach alphas: 0.68–0.79). Scores on each orientation scale ranged from 0 to 8 and are presented as percentages of a total possible out of 8. For example, 0 on the assimilation scale indicated that the respondent did not answer “the US” or “white-American culture” to any of the items which is the equivalent of 0%. An 8 indicated that the respondent answered “the US” or “white-American culture” to all eight items, which represents a score of 100%.

Household social position was measured using the Hollingshead's Two-Factor Index of Social Position [29]. The Hollingshead scale was used because it is one of the most commonly used socioeconomic measures. The Hollingshead score is a composite measure of educational attainment and occupational rank and was computed in the following manner. An education score (1 through 7, with 1 equal to less than a seventh-grade education and 7 equal to graduate training) and an occupation score (1 through 7, with 1 equal to unskilled employee and 7 equal to higher executives, proprietors of large businesses, and major professionals) were assigned for each parent/guardian based on information provided by them. Education and occupation scores were then weighted to obtain a single score for each parent/guardian (range of 8 to 49) that reflects one of five social strata (1 through 5, with 1 being a reference to unskilled laborers or low social position and 5 a reference to major professionals or high social position). For families with multiple caretakers, scores for each were averaged to obtain a single household social position score. Individuals whose primary activities were homemaking, school, or who received state assistance did not have categorizable occupations according to the Hollingshead method and were not included in the household social position score (*n* = 42). The Hollingshead method has relatively good interrater agreement (67–96%) [30] and correlates well with other measures of social position (*r* = 0.73–0.86) [30, 31]. 

### 2.2. Statistical Analyses

Data analysis included data summarization, Spearman correlations, and multivariate regression modeling. Data were evaluated for normality before analysis and natural log transformations were made when necessary. Our total sample size included 156 participants. Of the 156 participants, 20 participants were missing data for dietary intake, moderate-to-vigorous physical activity, and/or sedentary time and were not included in the multivariate regression analyses. Mean variable differences by ethnicity were analyzed by independent *t*-tests, Chi-square, and ANCOVA. All analyses were performed using SPSS 18.0 (SPSS, Inc., Chicago, IL).

Spearman correlations were used to explore the associations between sociocultural and socioeconomic variables (i.e., AHIMSA subscales, household social position) and FSIGT parameters (i.e., S_I_, AIR_G_, and DI). Multivariate regression models were used to further explore the relationships between the independent variables (i.e., AHIMSA subscales, household social position) and dependent variables (i.e., S_I_, AIR_G_, and DI). Specifically, partial correlations and parameter estimates were used to describe the relationship between sociocultural and socioeconomic variables and FSIGT variables after controlling for a priori covariates. A priori covariates included sex, Tanner stage, fat mass, fat-free mass, energy intake, moderate-to-vigorous physical activity, sedentary time, household social position (for sociocultural variables only), and S_I_ (for AIR_G_ only). Statistical analyses that addressed the objectives of this study were stratified by ethnicity for the reason that both qualitative and quantitative variables were examined in this study with African-Americans using a modified version of the AHIMSA questionnaire (i.e., response items were modified for assimilation and separation) and Latino-Americans using the standard version. A priori significance level was set at *P* < 0.05. All assumptions for multiple linear regression were satisfied and FSIGT variables were log transformed in order to meet these assumptions. Data reported are means ± SE.

## 3. Results

Table 2 displays the participant characteristics for the 43 African-American and 113 Latino-American boys and girls included in this study. For household social position, 60.4% of African-American households classified themselves in either the “middle” or “upper-middle” categories, compared to 17.7% for Latino-American households. For behavioral factors, African-American children and adolescents participated in significantly fewer minutes of moderate-to-vigorous physical activity per week, compared to Latino-Americans (*P* < 0.05); however, dietary patterns were similar across ethnic groups. For biological factors, African-American adolescents were significantly taller and heavier than their Latino-American counterparts, with higher BMIs, volumes of fat mass and fat-free mass (all *P*'s < 0.05). After controlling for sex, Tanner stage, fat mass, fat-free mass, and S_I_ (for AIR_G_ only), fasting glucose and S_I_ were significantly lower in African-American children and adolescents compared to Latino-Americans (both *P*'s < 0.01). In addition, HOMA, AIR_G_, and DI were significantly higher in African-Americans compared to Latino-Americans (all *P*'s < 0.05). Ethnic differences were not observed in age, Tanner stage, BMI percentile, total dietary intake, and sedentary time. 

Spearman correlations revealed that, for African-American children and adolescents, assimilation was positively associated with log S_I_ (*ρ* = 0.33, *P* < 0.05) and marginalization was positively associated with log AIR_G_ (*ρ* = 0.39, *P* < 0.05). However, these relationships were no longer significant in our multivariate regression analyses suggesting confounding of biological and behavioral covariates included in our model. For Latino-American children and adolescents, Spearman correlations revealed a negative association between integration and DI (*ρ* = −0.20, *P* < 0.05); again this relationship was no longer significant after controlling for biological and behavioral covariates in our regression analyses. Household social position was negatively associated with DI (*ρ* = −0.37, *P* < 0.05) in Latino-Americans; this relationship remained significant in our regression analyses. 

Table 3 displays the results of the multiple regression analysis for African-American children and adolescents. We observed a positive parameter estimate and partial correlation between the AHIMSA subscale integration, log AIR_G_ and log DI. Also, log AIR_G_ was positively associated with integration (*β* = 0.27 ± 0.09, *r* = 0.48, *P* < 0.01) after controlling for sex, Tanner stage, fat/fat-free mass, energy intake, moderate-to-vigorous physical activity, sedentary time, and S_I_. Additionally, log DI was also positively associated with integration (*β* = 0.28 ± 0.09, *r* = 0.55, *P* < 0.01), after controlling for covariates. From these results and with all confounding effects of covariates being equal, predicted mean AIR_G_ was 92% higher for African-American children and adolescents at the 75th compared to 25th percentile of the AHIMSA integration subscale. Predicted mean DI was 93% higher for African-American children and adolescents at the 75th compared to 25th percentile of the AHIMSA integration subscale. Although the bivariate analysis did not reveal a significant correlation between integration, log AIR_G_, and log DI, once other variables were accounted for, these relationships became significant, suggesting negative confounding by covariates in our model. There were no significant relationships between household social position, other AHIMSA subscales (i.e., separation, assimilation, and marginalization), and FSIGT parameters. 

Table 4 displays the results of the multiple regression analysis for Latino-American children and adolescents. We observed a negative parameter estimate and partial correlation between household social position, log AIR_G_, and DI. Moreover, log AIR_G_ was inversely associated with household social position (*β* = −0.010 ± 0.004, *r* = −0.19, *P* = 0.02), after controlling for sex, Tanner stage, fat/fat-free mass, energy intake, moderate-to-vigorous physical activity, sedentary time, and S_I_. In addition, DI was inversely associated with household social position (*β* = −20.44 ± 7.50, *r* = −0.27, *P* < 0.01), after controlling for biological and behavioral covariates. To better understand which of the two socioeconomic indicators measured was driving the inverse relationship between household social position and diabetes risk, we also calculated parameter estimates and partial correlations for educational attainment, occupational rank, log AIR_G_, and DI. These analyses revealed that log AIR_G_ and DI were significantly associated with parental educational attainment (AIR_G_: *β* = −0.09 ± 0.36, *r* = −0.19, *P* = 0.01; DI: *β* = −189.56 ± 61.72, *r* = −0.29, *P* < 0.01), whereas the associations between parental occupational rank, log AIR_G_, and DI were nonsignificant (data not shown). From these results and with all covariates being equal, predicted mean AIR_G_ was 129% lower for Latino-American children and adolescents at the 75th compared to 25th percentile of parental education. The model for DI contained an intercept of 3600 × 10^−4^ min^−1^ and a parameter estimate of −189.6 × 10^−4^ min^−1^ for every one-unit increase in parental education. From these results and with all covariates being equal, DI was 31% lower for Latino-American children and adolescents at the 75th compared to 25th percentile of parental education. There were no significant relationships between AHIMSA subscales and FSIGT parameters.

## 4. Conclusions

Pancreatic beta-cells have the ability to increase insulin secretion (via AIR_G_) in response to insulin resistance. This nonlinear hyperbolic relationship between sensitivity and secretion is best described as DI [25, 32]. Hence, higher AIR_G_ and DI typically represent an ability to compensate for insulin resistance in order to maintain normal glucose tolerance (i.e., lower diabetes risk). In contrast, lower AIR_G_ and DI represent an inability of the pancreas to secrete enough insulin at a given level of insulin resistance where impaired glucose tolerance may arise (i.e., higher diabetes risk). Indeed, our laboratory has shown both increased AIR_G_ and DI as potential compensatory mechanisms for decreased S_I_ in minority children and adolescents [3, 27]. The underlying determinants that contribute to increased insulin resistance and pancreatic beta-cell dysfunction in overweight/obese African-American and Latino-American children and adolescents are unknown; however sociocultural and socioeconomic factors each play a unique role in shaping diabetes risk in ethnic minorities. In the present analysis, the sociocultural adaptive style of combining aspects of both mainstream white-American culture while retaining aspects of their own family's culture was negatively associated with type 2 diabetes risk in overweight/obese African-American children and adolescents (as reflected by higher AIR_G_ and DI). These relationships remained significant after adjusting for household social position and other behavioral and biological covariates. In contrast, household social position was positively associated with type 2 diabetes risk in Latino-American children and adolescents (via decreased AIR_G_ and DI). Taken together, these findings suggest that sociocultural factors may be important predictors of type 2 diabetes risk in overweight/obese African-American children and adolescents whereas socioeconomic factors, rather than culture, may be more important for Latino-Americans.

African-Americans are a heterogeneous ethnic group who vary in the extent to which they both retain their black-American culture and also adopt aspects of white-American culture [33]. Previous research on adults has documented the relevance of these adaptive cultural styles to health and health-related behaviors in African-American adults [10, 34]. Dressler et al. [34] reported African-Americans living in accordance with culturally constructed local community norms—or “cultural consonance” in lifestyle—were a stronger independent predictor of smoking and hypertension than were indicators of socioeconomic position (i.e., occupation, income and education). Airhihenbuwa et al. [10] reported that positive identification with African-American culture and a self-perception of being successful in both the “black” and “white” ways of life were associated with healthy behaviors, including reduced fat consumption, more participation in leisure-time physical activity, reduced smoking, and, in women only, reduced alcohol consumption. Our results are generally consistent with these findings and suggest that the protective health effects of integrating two cultures also extend to overweight/obese African-American children and adolescents at increased risk for type 2 diabetes. In essence, integrating aspects of both black-American and white-American cultures was associated with lower diabetes risk (via increased AIR_G_ and DI), independent of household social position, physical activity, sedentary time, dietary intake, sex, Tanner stage, and fat/fat-free mass.

An association between culture and type 2 diabetes risk, independent of physical activity and diet, is plausible, given what is known about the physiological mechanisms linking psychosocial stress to insulin resistance and subsequent type 2 diabetes risk via hypothalamic-pituitary-adrenal axis activation [35, 36]. In general, integration of two or more cultures is viewed as a less stressful, more adaptive process, because this orientation allows ethnic minorities to function effectively in a multicultural society while still maintaining supportive connections to their own family's culture [37]. Hence, integration may be associated with lower psychological stress in African-Americans, thereby influencing type 2 diabetes risk independent of physical activity and diet. Additional research is needed to better understand the associations between integration, psychological stress, and diabetes risk in this ethnic minority group.

Many more researchers have investigated the influence of sociocultural factors on diabetes risk in Latino-Americans [12]. The influence of culture on behavior and subsequent diabetes risk is inconsistent [18] and may be confounded by socioeconomic position [19]. In the present study, household social position, not sociocultural orientation, was positively associated with type 2 diabetes risk in Latino-American children and adolescents. This relationship remained significant after controlling for biological and behavioral factors. Moreover, post hoc analyses revealed that, of the two socioeconomic indicators measured (educational attainment and occupational rank), parental education was driving the relationship between household social position and diabetes risk.

A protective effect of socioeconomic position and educational attainment in particular on type 2 diabetes risk has been well established among adults and non-Latino whites [21]; however, in the present study, this relationship was not present in either ethnic group. The rationale for the absent relationship in African-Americans and paradoxical relationship in Latino-Americans is unclear. Nevertheless similar findings have been previously reported between socioeconomic position and other metabolic outcomes in minority children and adolescents [38]. Using data from the National Health and Nutrition Examination Survey and National Health Interview Survey, Sobal and Stunkard [38] reported that ethnic minority children from higher socioeconomic households were just as likely to be overweight and obese as compared to children residing in lower socioeconomic households. These findings taken together with those in the present study suggest that residing in higher socioeconomic households may not be protective against obesity and subsequent type 2 diabetes risk in ethnic minority children and adolescents as has been previously reported in non-Latino whites. Moreover, parental education may be a stronger independent predictor of type 2 diabetes risk than culture in Latino-Americans; additional research is warranted.

Several limitations of this study should be noted. First, data limitations precluded analysis of other factors known to influence diabetes risk in this analysis including genetic admixture [39], smoking status and alcohol consumption [40], social desirability [41], and self-reported psychological stress [42]. Similarly, proxy indicators of acculturation such as language use, nativity, and time in the US were not available for our participants [14]. Second, although prior research suggests that household social position and sociocultural orientation are predictors rather than consequences of diabetes risk [10, 21, 34], the cross-sectional nature of this study impeded our ability to make causal inferences. Third, these findings in a small sample of overweight/obese African-American and Latino-American children and adolescents living in the Greater Los Angeles area cannot necessarily be generalized to all adolescents living in the US. Finally, post hoc power calculations revealed that some of our analyses were underpowered given the large variability in FSIGT-derived insulin and glucose indices. Despite being underpowered, we were able to detect significant associations between the AHIMSA subscale integration, AIR_G_, and DI in African-Americans as well as significant associations between household social position, AIR_G_, and DI in Latino-Americans. Thus, our findings may be an underestimation of the true effect of sociocultural orientation and household social position on type 2 diabetes risk in overweight/obese African-American and Latino-American children and adolescents. Nevertheless, additional research examining these relationships in a larger, more homogenous sample may better elucidate the role of sociocultural and socioeconomic factors in shaping type 2 diabetes risk in overweight/obese ethnic minority pediatric populations.

In summary, sociocultural orientation and household social position appear to play distinct and opposing roles in type 2 diabetes risk in overweight/obese African-American and Latino-American children and adolescents. For African-Americans, maintaining a sense of their own family's culture while integrating into mainstream white-American society was independently associated with decreased diabetes risk (as represented by increased AIR_G_ and DI). For Latino-Americans, increased diabetes risk was independently associated with increased household social position, higher parental education in particular, via decreased AIR_G_ and DI. Future research should continue to examine these factors over time to better understand the relationships between the sociocultural orientation, household social position, and type 2 diabetes risk in overweight/obese African-American and Latino-American children and adolescents. Moreover, behavioral interventions and public policies are needed to better address sociocultural and socioeconomic factors associated with type 2 diabetes risk in ethnic minority pediatric populations.

## Figures and Tables

**Table 1:** Acculturation, Habits, and Interests Multicultural Scale for Adolescents (AHIMSA) questionnaire and response items.

Questionnaire items	
“I am most comfortable being with people from…”	
“My best friends are from…”	
“The people I fit in with best are from…”	
“My favorite music is from…”	
“My favorite TV shows are from…”	
“The holidays I celebrate are from…”	
“The food I eat at home is from…”	
“The way I do things and the way I think about things are from…”	

African-American response items	

“White-American culture”	
“My family's culture”	
“Both”	
“Neither”	

Latino-American response items	

“The US”	
“The country my family is from”	
“Both”	
“Neither”	

Subscale definitions	

Assimilation—replacing my family's/native country's culture with that of white-American/mainstream American culture	
Separation—retaining my family's/native country's culture while rejecting white-American/mainstream American culture	
Integration—combining aspects of both cultures	
Marginalization—becoming alienated from both cultures	

**Table 2:** Participant characteristics.

	African-Americans	Latino-Americans	*P* value
Household social position	*n* = 43	*n* = 113	**<0.01**
Upper (%)	0	0	—
Upper-middle (%)	11.6	6.2	—
Middle (%)	48.8	11.5	—
Lower-middle (%)	23.3	34.5	—
Lower (%)	16.3	47.8	—
Behavioral factors	*n* = 39	*n* = 102	
Total dietary intake (kcal/day)	1898.7 ± 107.8	1870.2 ± 58.4	0.91
Moderate/vigorous physical activity (min/wk)	89.9 ± 13.9	137.1 ± 10.2	**0.02**
Sedentary time (min/wk)	216.6 ± 19.2	205.0 ± 13.3	0.56
Biological factors	*n* = 43	*n* = 113	
Female (%)	79.1	76.1	0.70
Age (years)	13.6 ± 0.5	13.0 ± 0.3	0.30
Tanner stage (%)			0.24
1	14.0	20.4	—
2	14.0	19.5	—
3	7.0	4.4	—
4	11.6	20.4	—
5	53.5	35.4	—
Height (cm)	158.9 ± 1.9	152.4 ± 1.6	**0.01**
Weight (kg)	83.1 ± 4.8	70.5 ± 2.7	**0.02**
BMI (kg/m^2^)	31.9 ± 1.4	28.6 ± 0.7	**0.03**
BMI percentile	94.0 ± 2.1	90.9 ± 1.5	0.26
Fat-free mass (kg)	51.5 ± 2.6	45.2 ± 1.4	**0.02**
Fat mass (kg)	34.0 ± 3.3	25.4 ± 1.5	**0.02**
FSIGT parameters	*n* = 43	*n* = 113	
Fasting glucose (mg/dL)	88.7 ± 1.0	92.1 ± 0.6	**<0.01**
Fasting insulin (*μ*IU/mL)	23.1 ± 4.1	19.1 ± 1.1	0.94
HOMA-IR	5.2 ± 0.9	4.4 ± 0.3	**<0.01**
S_I_ (×10^−4^ min^−1^/(*μ*IU/mL))	1.7 ± 0.2	2.4 ± 0.1	**<0.01**
AIR_G_ (*μ*IU/mL)	2200.5 ± 258.8	1256.8 ± 81.5	**<0.01**
DI (×10^−4^ min^−1^)	2587.7 ± 247.6	2110.0 ± 93.9	**0.05**

Data are Mean ± SE. Significant at *P* < 0.05. BMI: body mass index; FSIGT: frequently-sampled intravenous glucose tolerance test; S_I_: insulin sensitivity; AIR_G_: acute insulin response to glucose; DI: disposition index. *P* values were calculated using Chi-square (i.e., sex, Tanner stage, and household social position); Student's *t*-tests (i.e., age, anthropometry, dietary intake, physical activity and sedentary time) and analysis of covariance (i.e., glucose and insulin indices). Covariates included: sex, Tanner stage, fat/fat-free mass. While unadjusted means are reported here for all variables, analyses were based on log scores for age, fasting insulin, insulin sensitivity, acute insulin response, total dietary intake, assimilation, separation, integration, and marginalization.

**Table 3:** Results of multiple regression analysis for African-American children and adolescents (*n* = 34).

Outcome	Parameters	*β*_ (parameter)_	*r*_ (parameter)_	*P* value
	Household social position^††^	−0.01 ± 0.01	−0.14	0.44
	Integration^†^	0.06 ± 0.10	0.13	0.56
log HOMA-IR	Separation^†^	0.09 ± 0.08	0.20	0.27
	Assimilation^†^	−0.04 ± 0.12	−0.10	0.72
	Marginalization^†^	0.02 ± 0.11	0.03	0.89

	Household social position^††^	0.003 ± 0.007	0.05	0.70
	Integration^†^	0.05 ± 0.07	0.12	0.45
log S_I_	Separation^†^	−0.03 ± 0.05	−0.07	0.63
	Assimilation^†^	−0.04 ± 0.08	−0.11	0.63
	Marginalization^†^	−0.10 ± 0.07	−0.22	0.19

	Household social position^††^	−0.01 ± 0.01	−0.12	0.48
	Integration^†^	0.27 ± 0.09	**0.48**	**<0.01**
log AIR_G_	Separation^†^	−0.03 ± 0.09	−0.06	0.71
	Assimilation^†^	−0.07 ± 0.13	−0.14	0.59
	Marginalization^†^	0.08 ± 0.13	0.13	0.55

	Household social position^††^	−0.01 ± 0.01	−0.11	0.52
	Integration^†^	0.28 ± 0.09	**0.55**	**<0.01**
log DI	Separation^†^	−0.04 ± 0.09	−0.08	0.65
	Assimilation^†^	−0.08 ± 0.13	−0.18	0.53
	Marginalization^†^	0.04 ± 0.12	0.08	0.73

S_I_: insulin sensitivity; AIR_G_: acute insulin response to glucose; DI: disposition index. ^†^Models control for: Tanner stage, sex, fat mass, log fat-free mass, log energy intake, log moderate-to-vigorous physical activity, log sedentary time, and household social position. ^††^Model controls for: Tanner stage, sex, fat mass, log fat-free mass, log energy intake, and log moderate-to-vigorous physical activity and log sedentary time. For log AIR_G_ models, S_I_ was included as a covariate.

**Table 4:** Results of multiple regression analysis for Latino-American children and adolescents (*n* = 102).

Outcome	Parameters	*β*_ (parameter)_	*r*_ (parameter)_	*P* value
	Household social position^††^	0.002 ± 0.005	0.04	0.60
	Integration^†^	0.05 ± 0.05	0.10	0.29
log HOMA-IR	Separation^†^	0.02 ± 0.04	0.05	0.57
	Assimilation^†^	−0.04 ± 0.04	−0.08	0.36
	Marginalization^†^	0.06 ± 0.05	0.11	0.18

	Household social position^††^	−0.004 ± 0.005	−0.08	0.33
	Integration^†^	−0.01 ± 0.05	−0.02	0.82
log S_I_	Separation^†^	0.00 ± 0.04	0.00	0.99
	Assimilation^†^	−0.03 ± 0.04	−0.07	0.41
	Marginalization^†^	−0.07 ± 0.05	−0.13	0.10

	Household social position^††^	−0.010 ± 0.004	−**0.19**	**0.02**
	Integration^†^	0.02 ± 0.05	0.03	0.70
log AIR_G_	Separation^†^	−0.06 ± 0.03	−0.13	0.08
	Assimilation^†^	−0.02 ± 0.04	−0.05	0.52
	Marginalization^†^	0.04 ± 0.043	0.07	0.36

	Household social position^††^	−20.44 ± 7.50	−**0.27**	**<0.01**
	Integration^†^	28.62 ± 80.97	0.04	0.73
DI	Separation^†^	−50.75 ± 58.38	−0.08	0.39
	Assimilation^†^	−57.85 ± 61.29	−0.09	0.35
	Marginalization^†^	48.22 ± 75.23	0.06	0.52

S_I_: insulin sensitivity; AIR_G_: acute insulin response to glucose; DI: disposition index. ^†^Models control for: Tanner stage, sex, fat mass, log fat-free mass, log energy intake, log moderate-to-vigorous physical activity, log sedentary time, and household social position. ^††^Model controls for: Tanner stage, sex, fat mass, log fat-free mass, log energy intake, and log moderate-to-vigorous physical activity and log sedentary time. For log AIR_G_ models, S_I_ was included as a covariate.
